# Calcinose pseudo-tumorale primitive chez l’enfant

**DOI:** 10.11604/pamj.2017.28.239.11313

**Published:** 2017-11-16

**Authors:** Abdoulaye Diallo Harouna, Karima Atarraf, Abderrahmane My Afifi

**Affiliations:** 1 Service de Traumato-Orthopédique Pédiatrique, CHU Hassan II, Fès, Maroc; 2 Université Sidi Mohamed Ben Abdellah, Faculté de Médecine et de Pharmacie de Fès, Maroc

**Keywords:** Calcinose tumorale primitive, masse du tissu mou, enfant, Primary tumoral calcinosis, soft tissue mass, child

## Abstract

La calcinose pseudo-tumorale est une tumeur bénigne relativement rare, elle se caractérise par un dépôt de matériel calcique dans les tissus mous péri-articulaires. Nous rapportons un cas de calcinose tumorale primitive de la hanche gauche découvert chez un enfant de 15 ans avec une revue de la littérature. La patiente a été opérée avec une exérèse totale de la masse, les suites postopératoires étaient simples. 6 mois après l’intervention il n’y a pas de récidive tumorale.

## Abstract (en)

Pseudotumoral calcinosis is a relatively rare benign tumor, characterized by calcium deposition in peri-articular soft tissues. We here report the case of a 15-year old boy with primary tumoral calcinosis of the left hip as well as a literature review. The patient underwent total mass resection. The postoperative course was simple. The patient showed no recurrence at 6-month follow-up.

## Introduction

La calcinose pseudo-tumorale, est une tumeur bénigne rare, caractérisée par un dépôt de matériel calcique dans les tissus mous péri-articulaires prenant l'aspect d'une véritable tumeur [1]. Elle se présente sous deux formes cliniques: la forme sporadique, secondaire à une affection chronique (insuffisance rénale chronique, hyper-parathyroidisme…etc) [2,3] et la forme primitive dite familiale d'origine probablement génétique [4,5]. A la date d'aujourd'hui, seulement de 100 cas de formes primitives ont été rapportés à travers la littérature [4].

## Patient et observation

**Figure 1:**
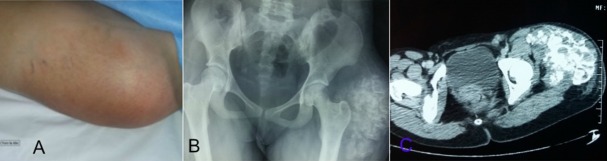
A) tuméfaction de la face antero-externe de la hanche gauche arrivant jusque au 1/3 supero-externe de la cuisse; B) radiographie du bassin en incidence de face montrant des opacités calcifiées, séparées par des septas radio-transparents péri-articulaire au niveau de la hanche gauche; C) tomodensitométrie montrant une masse en regard de l’articulation coxo-fémorale gauche, de densité calcique, bourgeonnante rappelant un aspect en chou-fleur

**Figure 2:**
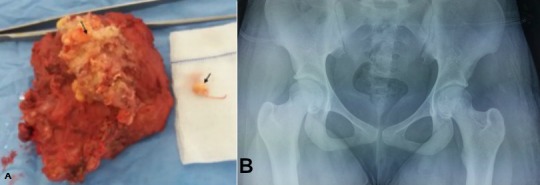
A) pièce d’exérèse de la tumeur; B) radiographie du bassin après 6 mois de l’intervention montre une disparition complète des dépôts calciques

T. I. est une patiente de 15 ans, suivie pour une ostéomyélite chronique des 2 pieds, elle était victime il y’a un an d’un traumatisme de la hanche gauche suite à une chute de sa hauteur. La patiente n'avait aucun antécédent familial particulier. Elle était admise pour une tuméfaction indolore de la hanche gauche d'évolution progressive depuis 8 mois, sans notion de fièvre ni de boiterie, avec conservation de l'état général. L'examen clinique notait une volumineuse masse en regard de l'articulation coxo-fémorale gauche mesurant 15cm sur 10cm de dimensions, elle s'étend jusqu'au 1/3 moyen de la face externe de la cuisse (Figure 1 A). Cette masse était de consistance ferme, fixée par rapport au plan profond et mobile par rapport à la peau, sans signes inflammatoires en regard, le reste de l'examen ostéoarticulaire était sans anomalie. La radiographie du bassin en incidence de face avait montré une masse calcifiée péri-articulaire, coiffant le grand trochanter sans lyse de celui-ci (Figure 1 B). L'étude échographique de cette masse révélait un aspect multi-loculé, hétérogène renfermant des calcifications. Le complément tomodensitométrique (Figure 1 C) précisait la lésion sus décrite en confirmant l'absence d'envahissement ostéo-articulaire. La masse avait une densité calcique bourgeonnante en chou-fleur au sein desquelles existent des logettes hypo-denses en faveur d'une calcinose pseudo-tumorale. Il n'y avait pas de trouble phosphocalcique biologique. Devant cette énorme masse une chirurgie à viser diagnostique et thérapeutique était jugée nécessaire. La voie d'abord était une incision de Hueter et l'exploration trouvait une masse calcifiée ayant un contact intime avec le grand trochanter sans l'envahir. Il s'y associait un refoulement de tous les plans musculaires adjacents à la masse. La masse a été totalement reséquée en suivant les plans de clivage entre celle-ci et les plans musculaires. L'analyse histologique de la pièce d'exérèse (Figure 2 A) avait objectivé: un tissu musculo-adipeux dissocié par de larges plages de calcifications de taille et de formes variées, cernées par une réaction histiocytomacrophagique gigantocellulaire à corps étranger sans lésion suspecte de malignité concluant a une calcinose musculaire. Les suites opératoires furent simples, avec un recul de 6mois, la patiente n'a aucune plainte particulière ni de récidive locale au dernier contrôle radiologique (Figure 2 B).

## Discussion

La calcinose pseudo-tumorale est une tumeur bénigne rare, seulement près de 100 cas de formes primitives étaient rapportés à travers la littérature [4]. Elle est caractérisée par un dépôt de matériel calcique dans les parties molles juxta-articulaires [5,6]. Si dans les formes secondaires (insuffisants rénaux chroniques hémodialysés) le dépôt calcique serait lié à une anomalie du métabolisme phosphocalcique [2,3], dans les formes primitives, le mécanisme est encore moins clair. Il serait probablement d'origine génétique par transmission autosomique récessive [1], responsable d'une hyperphosphoremie familiale par perturbation enzymatique sans insuffisance rénale associée [7]. Chez notre malade, aucune une histoire familiale n'avait été retrouvée et elle n'avait pas d'insuffisante rénale non plus, ce qui constitue une description rare de la maladie. Certains facteurs favorisants sembles être en cause: des traumatismes ou microtraumatismes seraient à l'origine d'une ischémie locale qui déclencherait une réaction inflammatoire responsable de dépôt de matériel phosphocalcique) [5,8]. En effet, selon Thomson qui avait étudié la survenue de la maladie chez une population noire africaine, dormir sur plan dur expose particulièrement la région fessière, le grand trochanter, l'épaule et le coude à la survenue de la calcinose tumorale, probablement par microtraumatismes[9]. Ce mécanisme pourrait probablement être une piste à privilégier pour tenter d'expliquer la survenue des dépôts calciques chez les patients sans antécédents familiaux ni maladies chroniques comme le cas actuel notre patiente qui avait justement un antécédent de traumatisme précèdent la symptomatologie. La calcinose familiale touche particulièrement les patients entre la première et deuxième décade sans prédominance de sexe, notre patiente se trouve dans la deuxième décade, la race noire semble plus exposée [6,9]. La calcinose pseudo-tumorale se caractérise par une tuméfaction de taille plus ou moins impressionnante, allant de 1à 30cm de diamètre [5] autour des grosses articulations le plus souvent, en particulier la région trochantérienne, comme dans le cas de notre malade. Cliniquement la calcinose pseudo-tumorale se présente comme une tumeur de taille et de croissance variables, le plus souvent asymptomatique, elle peut devenir symptomatique dans certaines localisations et peut devenir douloureuse par compression des structures avoisinantes ou gène fonctionnelle, avec limitation du périmètre de marche, comme il comme l'exemple de notre cas précis. Dans la plupart des cas le diagnostic préopératoire n'est pas toujours évident, avec la hantise pour tout chirurgien de me connaitre une tumeur maligne. Le bilan biologique est souvent normal comme dans le cas de notre patiente [8]. La radiographie standard évoque le diagnostic en montrant des calcifications juxta articulaire [7,10] réalisant un aspect en grappe par juxtaposition des petites images denses, arrondies et bien limitées homogènes, séparées par des septas radio-transparents[7] ,cette calcification se développait par sédimentation réalisant une masse le plus souvent hétérogène d'image classique en nid d'abeille [5], tous ces caractères ont été retrouvés chez notre malade. La TDM voire l'IRM permet de mieux caractériser la lésion et de préciser les rapports avec les structures musculo-aponévrotiques et osseuses [5]. Toute calcinose pseudo tumorale devenant symptomatique par son volume et ou sa localisation, l'exérèse chirurgicale complète est la règle [1]. Le traitement chirurgical consiste à une exérèse totale de la lésion garant du non récidive tumorale. Le diagnostic définitif est histopathologique à travers une biopsie chirurgicale par crainte de méconnaitre une tumeur maligne. Le traitement est chirurgical avec exérèse totale. Toute exérèse incomplète est pourvoyeuse de récidive tumorale. Les suites opératoires sont généralement simples et le pronostic reste bon.

## Conclusion

La calcinose pseudo-tumorale primitive est une pathologie bénigne rare, qui nécessite tout de même une enquête étiologique approfondie afin d'écarter une malignité et de rechercher et traiter une cause sous-jacente la seule garantie d'une guérison sans récidive. L'exérèse chirurgicale est la seule alternative devant toute calcinose pseudo tumorale symptomatique.

## Conflits d’intérêts

Les auteurs ne déclarent aucun conflit d'intérêts.
